# Off-axis digital lensless holographic microscopy based on spatially multiplexed interferometry

**DOI:** 10.1117/1.JBO.29.S2.S22715

**Published:** 2024-08-19

**Authors:** José Ángel Picazo-Bueno, Steffi Ketelhut, Jürgen Schnekenburger, Vicente Micó, Björn Kemper

**Affiliations:** a University of Muenster, Biomedical Technology Center, Muenster, Germany; b University of Valencia, Department of Optics, Optometry and Vision Science, Burjassot, Spain

**Keywords:** quantitative phase imaging, off-axis lensless holography, digital lensless holographic microscopy, label-free imaging, digital holographic microscopy, phase retrieval, spatially multiplexed interferometric microscopy

## Abstract

**Significance:**

Digital holographic microscopy (DHM) is a label-free microscopy technique that provides time-resolved quantitative phase imaging (QPI) by measuring the optical path delay of light induced by transparent biological samples. DHM has been utilized for various biomedical applications, such as cancer research and sperm cell assessment, as well as for *in vitro* drug or toxicity testing. Its lensless version, digital lensless holographic microscopy (DLHM), is an emerging technology that offers size-reduced, lightweight, and cost-effective imaging systems. These features make DLHM applicable, for example, in limited resource laboratories, remote areas, and point-of-care applications.

**Aim:**

In addition to the abovementioned advantages, in-line arrangements for DLHM also include the limitation of the twin-image presence, which can restrict accurate QPI. We therefore propose a compact lensless common-path interferometric off-axis approach that is capable of quantitative imaging of fast-moving biological specimens, such as living cells in flow.

**Approach:**

We suggest lensless spatially multiplexed interferometric microscopy (LESSMIM) as a lens-free variant of the previously reported spatially multiplexed interferometric microscopy (SMIM) concept. LESSMIM comprises a common-path interferometric architecture that is based on a single diffraction grating to achieve digital off-axis holography. From a series of single-shot off-axis holograms, twin-image free and time-resolved QPI is achieved by commonly used methods for Fourier filtering-based reconstruction, aberration compensation, and numerical propagation.

**Results:**

Initially, the LESSMIM concept is experimentally demonstrated by results from a resolution test chart and investigations on temporal stability. Then, the accuracy of QPI and capabilities for imaging of living adherent cell cultures is characterized. Finally, utilizing a microfluidic channel, the cytometry of suspended cells in flow is evaluated.

**Conclusions:**

LESSMIM overcomes several limitations of in-line DLHM and provides fast time-resolved QPI in a compact optical arrangement. In summary, LESSMIM represents a promising technique with potential biomedical applications for fast imaging such as in imaging flow cytometry or sperm cell analysis.

## 1 Introduction

Digital holographic microscopy (DHM) is a label-free microscopy technique providing accurate and time-resolved quantitative phase information of transparent biological samples.^1^^–^^3^ In contrast to other label-free techniques such as bright field, Zernike phase contrast,^4^ and differential interference contrast (DIC) microscopy,^5^ DHM quantitatively recovers the optical path delay of the light passing through semi-transparent samples.^6^^,^^7^ This enables quantitative phase imaging (QPI) with up to a nanometer sensitivity and extended depth of field, employing a non-invasive, full-field, real-time capable, non-contact, and static (without mechanical parts) operational principle.^8^^–^^11^ In earlier studies, various biomedical applications of DHM^3^^,^^12^^,^^13^ were demonstrated; these include cancer research,^14^^–^^16^ *in vitro* cytotoxicity testing,^17^^–^^19^ cell manipulation monitoring,^20^ immune cell analysis,^21^^–^^23^ sperm cell assessment,^24^^,^^25^ detection of viral infections,^26^ diabetes screening,^27^ and SARS-Cov-2 detection and classification.^28^

Digital lensless holographic microscopy (DLHM) is a variant of DHM and offers a simple and compact microscopy scheme with high resolution and a wide field of view (FOV)^29^ by implementing a digital version of Gabor’s initial holography approach,^30^ in which a point source illuminates the sample in transmission and a digital sensor records the diffraction pattern.^31^ Then, the sample image is recovered by numerical propagation from the recording/hologram plane to the image plane.^32^ However, the initially proposed in-line holographic configuration causes an overlap between the numerically focused image and the unfocused diffraction pattern of the conjugate complex twin image. This overlap not only affects the quality of the final image by reducing the signal-to-noise ratio (SNR) but also prevents an accurate acquisition of QPI images.^29^

Various approaches have been developed to address the twin-image problem in DLHM.^33^^–^^49^ These approaches include phase-shifting techniques^33^^,^^34^ and multi-height phase-retrieval procedures,^35^^,^^42^^–^^49^ which require recording multiple holograms to eliminate the twin image contribution. However, these earlier reported methods are limited or not suitable for fast dynamic events due to the need for multiple hologram recordings. Alternatively, single-shot techniques, which allow for twin-image-free image reconstruction from a single hologram, have been devised.^36^^–^^41^ These methods typically rely on iterative phase-retrieval algorithms with object mask constraints^36^^,^^37^ or multi-wavelength illumination.^38^^–^^41^ Although effective in mitigating the twin image, iterative algorithms can be computationally intensive and prone to convergence issues.^50^ Other approaches involved deep learning, which showed promising results in rapidly removing the twin image for specific applications, although they required training the algorithm with a substantial amount of suitable ground-truth data.^51^^–^^55^

An alternative approach to overcoming the twin-image problem in DLHM involves adopting an off-axis DLHM configuration.^56^^–^^59^ The off-axis approach allows for image reconstruction from single captured holograms without twin image presence and without a requirement for recording multiple images, iterative algorithms, object constraints, multi-illumination sources, or machine learning. For example, Lu et al.^56^ utilized an off-axis DLHM setup using two pinholes to achieve an interferometric configuration. Serabyn et al.^57^ employed a pair of small gradient-index (GRIN) lenses for off-axis holographic recording. Rostykus and Moser^58^ proposed the use of a prism with a photopolymer layer to record two-volume hologram gratings, enabling the implementation of an off-axis lensless configuration. In addition, Ebrahimi et al.^59^ presented a common-path off-axis DLHM scheme that utilizes a Fresnel biprism to generate two spherical waves for off-axis holographic recordings.

Here, we report on a common-path off-axis DLHM approach entitled LEnslesS spatially multiplexed interferometric microscopy (LESSMIM) to achieve both, single-shot QPI and twin-image elimination. LESSMIM is inspired by a previously reported DHM technique known as spatially multiplexed interferometric microscopy (SMIM),^60^^–^^63^ which extended a bright field microscope to a holographic one by introducing coherent illumination, leaving a clear region at the sample plane, and incorporating a diffraction grating. SMIM has been applied for super-resolution imaging,^64^^,^^65^ noise-reduced QPI,^61^^,^^66^ opposed-view QPI,^67^ and multimodal imaging.^68^ In this study, the application of SMIM is expanded to the field of DLHM-based QPI. Hence, LESSMIM implements a common-path off-axis interferometric configuration by assembling an illumination unit including a one-dimensional (1D) diffraction grating and by spatially multiplexing the sample plane. From a recorded off-axis hologram, LESSMIM achieves QPI by Fourier filtering-based reconstruction, aberration compensation, and numerical propagation.

Section 2 describes the concept and layout of our approach. Section 3 presents the experimental validation of LESSMIM using technical test targets, microspheres, and living cells. Experimental validations with living cells comprise evaluations with adherent cells having different sizes as well as evaluations with cells in flow using a microfluidic system with hydrodynamic focusing. Finally, Sec. 4 presents the discussion and conclusions of the proposed technique.

## 2 LESSMIM Concept and Experimental Setup

**Fig. 1:**
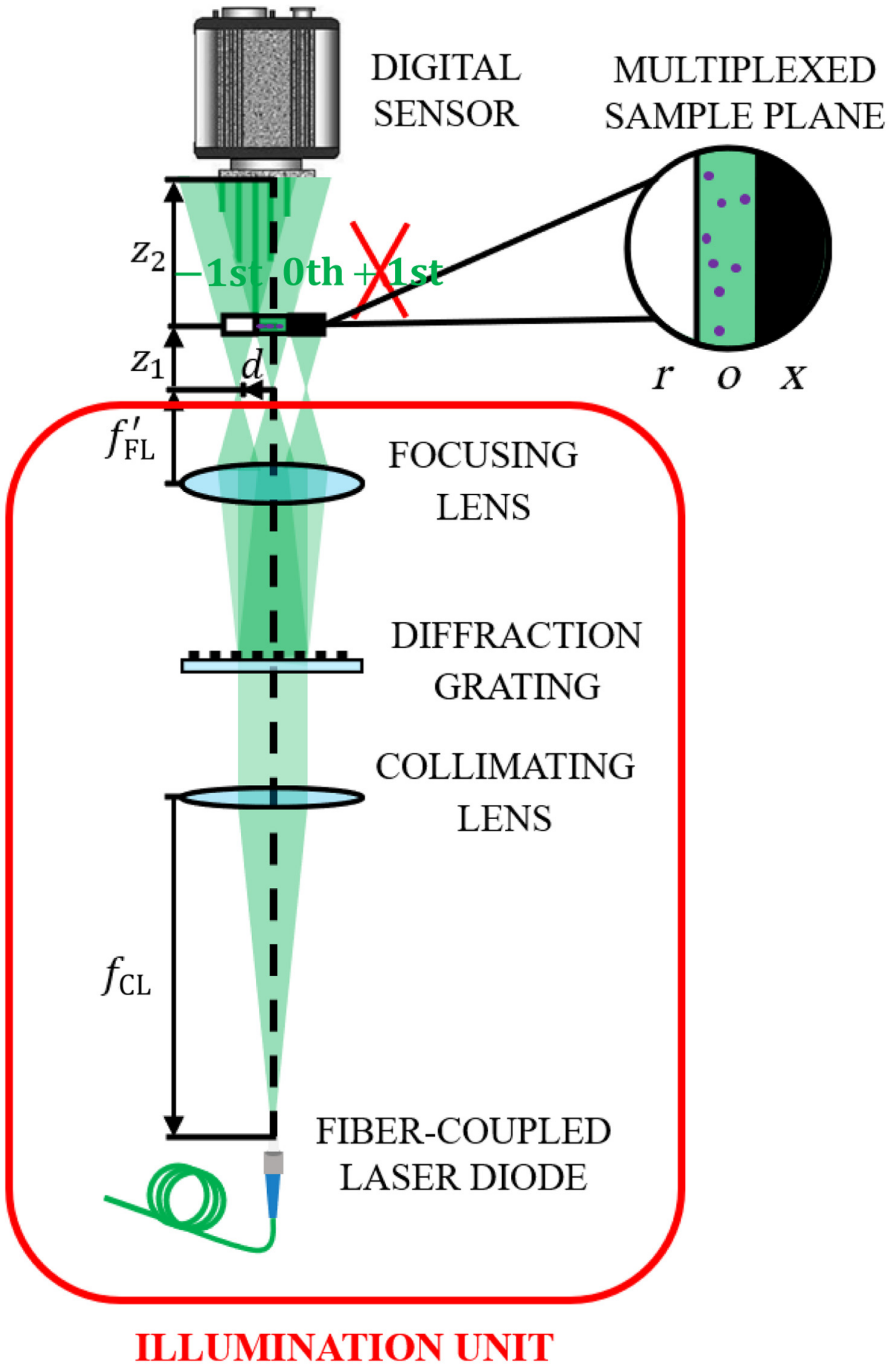
Scheme of the LESSMIM layout consisting of an illumination unit and a digital recording sensor. $f_{\mathrm{CL}}$ is the focal length of the collimating lens for object illumination with laser light, $f_{\mathrm{FL}}^{\prime }$ is the focal length of the focusing lens for the creation of three-point sources, $z_{1}$ is the distance between the point light sources and sample, $z_{2}$ is the distance between the sample and digital sensor, o is the object region, r is the reference region, and x is the blocking region.

Figure 1 illustrates the optical concept of LESSMIM, consisting of an illumination unit and a digital recording sensor. Collimated coherent illumination is achieved by a fiber-coupled laser diode applying a collimating lens with the focal length $f_{\mathrm{CL}}$. A sinusoidal diffraction grating diffracts the illumination wave into three fractions, which are focused by an additional lens with an image focal length $f_{\mathrm{FL}}^{\prime }$ to generate three spatially separated point sources in the focal plane. These replicas are laterally separated a distance

$$d=N\lambda f_{\mathrm{FL}}^{\prime },$$ (1)

where N is the spatial frequency of the grating, defined as the inverse of the spatial period of the grating periodic structures, and λ represents the wavelength of the illuminating laser light.^69^ The three resulting point sources, which correspond to the three diffraction orders of the grating, are located at a distance $z_{1}$ from the sample plane. The sample plane is divided into three regions from which one is blocked [object (o), reference (r), and blocking (x)], similar to previously reported SMIM implementations.^60^^,^^63^^,^^65^ The blocking region prevents spurious interferences, whereas the object and reference regions are areas with and without the sample for simultaneous object and reference wave transmission, respectively. The arrangement of these regions is shown in the upper panel of Fig. 1. The blocking region can be achieved in different ways: either a customized chamber with an opaque region is fabricated, a limiting aperture is placed at the plane where the point sources are generated to block one of the point sources, or a border of the opaque frame of the diffraction grating is used to block only a portion (one third) of the beam to produce the blocking effect. The most general use case to provide the blocking region is using an opaque border of the grating, but the choice of the different blocking options will depend on the experimental requirements of the specific setup. A digital sensor is positioned at a distance $z_{2}$ from the sample plane to record an off-axis digital hologram. The recorded hologram results from the coherent overlapping between the Fresnel diffraction pattern from the o region (0th diffraction order) and the non-perturbed light from the sample free r region (−1st order). Further details about the system alignment are provided in Sec. S1 of the Supplemental Material. Due to the divergence of the sample illumination, the recorded diffraction pattern of the object region is geometrically magnified with magnification M according to the relation^70^

$$M=(z_{1}+z_{2})/z_{1}.$$ (2)

The field of view (FOV) is determined by the size of the CMOS recording area and magnification as

$$\mathrm{FOV}={\left(Lp/M\right)}^{2},$$ (3)

where L is the number of pixels in each transversal direction and p is the pixel pitch of the digital sensor.^70^ The lateral resolution ρ is diffraction-limited and depends on the distance between the sample and sensor as well as on the sensor size as follows:^70^

$$\rho =2\lambda z_{2}/Lp.$$ (4)

The experimental validation of the LESSMIM concept was performed by utilization of a single-mode fiber-coupled laser diode (Thorlabs LP515-SF3, output power 3 mW, central wavelength 515 nm) as the coherent illumination source. An achromatic collimating lens (Linos, AC focal length $f_{\mathrm{CL}}=100\;\mathrm{mm}$) was used for collimation of the illuminating laser light, and a 20×/0.40 NA microscope lens (ZEISS LD Achroplan) was employed as the lens for beam focusing. Note that a specific microscope objective was used in the experimental validation of our concept, but more cost-effective optical lenses can be utilized. A Ronchi ruling (Edmund Optics, spatial frequency 120 line pairs/mm) served as a diffraction grating to generate the three-point sources. To generate the blocking region, we blocked one third of the beam with one opaque border of the Ronchi ruling frame. A monochrome CMOS sensor (TheImagingSource DMK23UP1300, 1280×1024  pixels, 4.8  μm pixel size, image acquisition rate 95 fps) was employed for recording the generated digital off-axis holograms. Holograms were transferred to a computer via a USB 3.0 interface for numerical reconstruction and image processing downstream using MATLAB 2021b. Despite the rectangular shape of the digital sensor, a squared region of interest (ROI) of 1024×1024  pixels was defined to achieve an equal lateral resolution in the x and y directions, according to Eq. (4). The sample was positioned at a distance of $z_{1}=1.0\pm 0.1\;\mathrm{mm}$ from the point source and $z_{2}=10.0\pm 0.1\;\mathrm{mm}$ from the digital sensor. The entire setup had a length of 200 mm from the optical fiber tip to the digital sensor. According to Eq. (2), a magnification of M=11× was set, resulting in an FOV of $447\times 447\;\mu m^{2}$, considering Eq. (3). Considering the above described optical and geometrical system parameters and Eq. (4), the theoretical spatial resolution is ρ=2.16±0.02  μm.

## 3 Experimental Validation

### 3.1 Demonstration of the Operation Principle with a Resolution Target

**Fig. 2:**
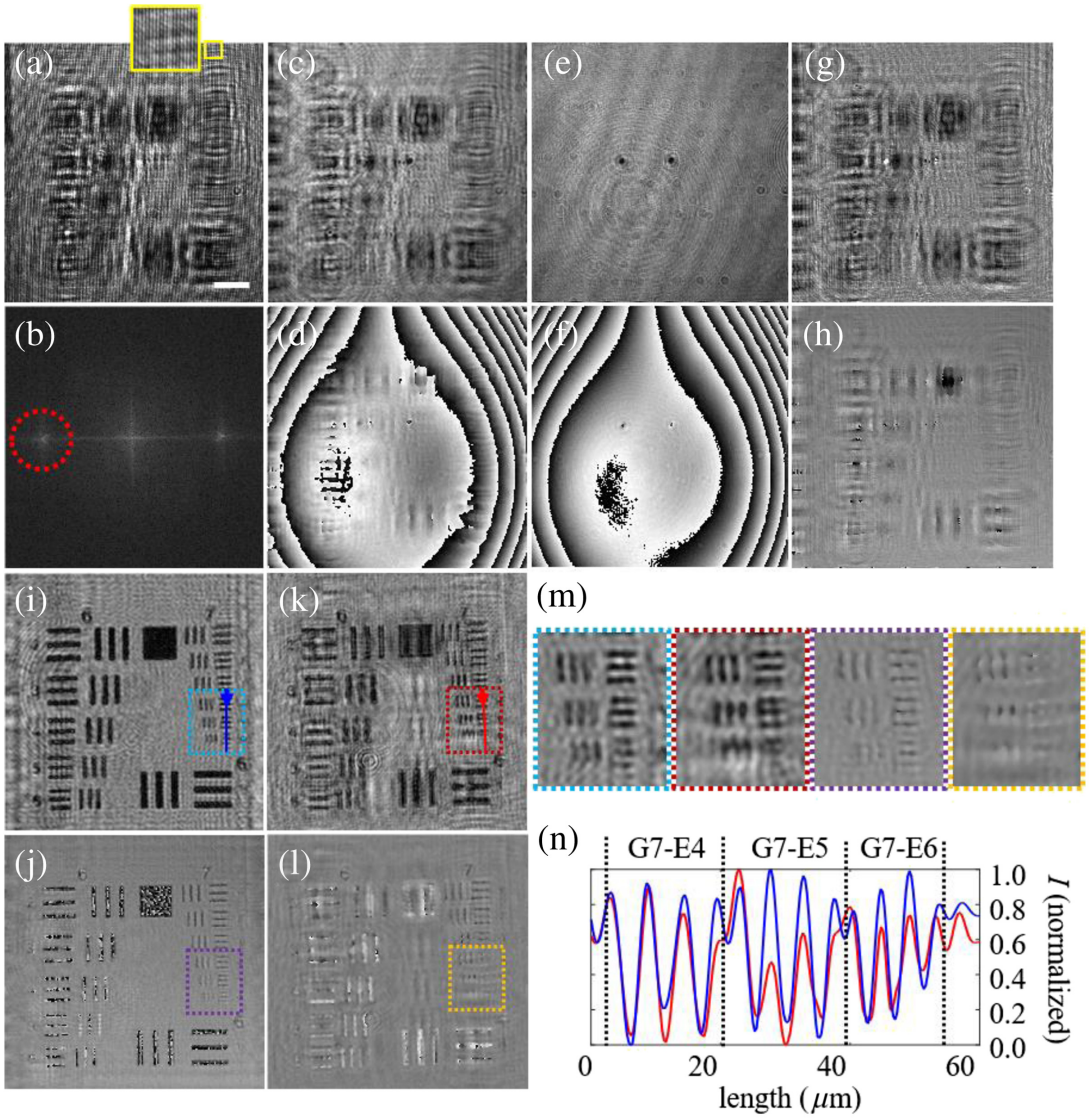
Illustration of the LESSMIM working principle for the example of a USAF resolution test target: (a) recorded off-axis hologram; (b) Fourier transformation of panel (a) with the filtered spectral region (marked with a dotted red circle); (c) amplitude and (d) phase reconstructions after Fourier filtering application; (e) amplitude and (f) phase reconstructions of the reference hologram; (g) amplitude and (h) phase images after subtraction of the amplitude and phase distributions retrieved from the reference hologram; (i) amplitude and (j) phase images after numerical propagation to the focus plane; and (k) amplitude and (l) phase images recovered by conventional in-line DLHM without a separate reference wave for direct comparison; (m) color-coded magnified images of regions in panels (i)–(l) containing the smallest elements of the resolution target; and (n) intensity profiles along blue and red lines in panels (i) and (k), respectively. The scale bar in panel (a) corresponds to a length of 20  μm.

In the first step, the operation principle of LESSMIM was evaluated. For that, off-axis holograms from a resolution test target (positive 1951 USAF target, Thorlabs Inc., New Jersey, United States) were recorded. Figure 2 illustrates the subsequently performed numerical reconstruction process. Figure 2(a) shows a recorded off-axis Fresnel digital hologram with an included magnified inset depicting the generated parallel off-axis carrier fringe pattern. Figure 2(b) displays the corresponding well-separated site bands of the diffraction orders in the spatial frequency domain after a fast Fourier transformation. Fourier filtering was applied to one of the cross-correlation terms, as indicated with a dotted red circle in Fig. 2(b), to retrieve the complex object wave, as well as the corresponding amplitude [Fig. 2(c)] and phase [Fig. 2(d)] distributions of the diffracted light. The reconstructed phase image in Fig. 2(d) exhibits a comatic aberration. This aberration was generated by the propagation of the tilted reference wave across the focusing lens through a region outside the optical axis. To compensate for the comatic effect, an additional blank reference hologram of a clear region of the USAF test target without sample information was recorded. Alternatively, it is also possible to acquire a reference hologram without a sample in the optical path. The retrieved amplitude and phase distributions are included in Figs. 2(e) and 2(f). Subsequently, the retrieved complex waves with [Figs. 2(c) and 2(d)] and without [Figs. 2(e) and 2(f)] sample information, including amplitude and phase information, were subtracted by complex division of the object wave and the wave from the blank reference hologram. As evident in Figs. 2(g) and 2(h), the subtraction process efficiently removed aberrations, spurious reflections, and dust, resulting in a homogeneous image background. The further numerical propagation of the subtracted complex object wave to the image plane was performed utilizing the angular spectrum method^71^ yielding focused amplitude [Fig. 2(i)] and phase [Fig. 2(j)] images of the test chart structures. To compare LESSMIM with conventional DLHM, amplitude and phase images obtained by DLHM were recovered as follows: the diffraction grating was simply removed to record a conventional DLHM hologram without a separate reference wave. The reconstruction of the conventional hologram was performed by numerical propagation as described in Ref. 71, and a reference hologram without a sample was recorded and employed for achieving a homogeneous image background in the amplitude and phases images^70^ [Figs. 2(k) and 2(l)]. In both DLHM images [Figs. 2(k) and 2(l)], disturbances by twin-image presence are evident, whereas the images provided by LESSMIM [Figs. 2(i) and 2(j)] exhibit a considerably higher contrast. The quality improvement of the amplitude and phase images achieved by LESSMIM is also indicated by the enlarged color-coded areas shown in Fig. 2(m) [see also blue and yellow rectangular frames in Figs. 2(i)–2(l)], in which the smallest elements of the applied USAF resolution test target are resolved (element 6 - group 7, G7-E6, period 4.38  μm). These observations are also supported by the cross-section profiles through the amplitude images [Fig. 2(n)] along the blue and red lines included in Figs. 2(i) and 2(k). Due to the smallest available structures of the applied USAF test target, the confirmed spatial resolution is lower than the theoretical value ρ=2.16  μm calculated in Sec. 2. For a more precise verification of the maximum achievable spatial resolution, a calibrated high-resolution USAF target, including group 8, element 6, which corresponds to a period of 2.19  μm, is required.

### 3.2 Temporal Stability

**Fig. 3:**
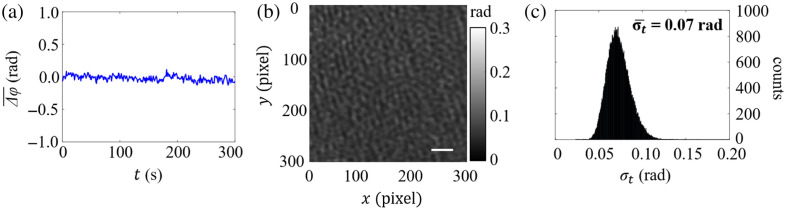
Temporal phase stability of LESSMIM during a period of 300 s within an ROI of 300×300  pixels utilizing a blank microscope slide as the sample location (hologram acquisition rate of 1 Hz). (a) temporal (t) development of the averaged phase $\overset{¯}{\Delta \varphi (t)}$; (b) spatial distribution of the standard deviation $\sigma_{t}(m,n)$ of the phase differences per pixel determined from the entire stack of 300 QPI images; and (c) histogram of the $\sigma_{t}$ from the data in panel (b). The mean value $\overset{¯}{\sigma_{t}}=0.07\;\mathrm{rad}$ quantifies a high temporal stability of the setup. The scale bar in panel (b) corresponds to 20  μm.

The temporal stability of LESSMIM was assessed by measuring phase fluctuations over time. Therefore, a blank microscope slide was placed at the sample location, and 300 off-axis holograms were sequentially recorded over a period of 5 min at a hologram acquisition rate of 1 Hz. After numerical QPI image reconstruction, within an ROI of 300×300  px, the variation $\overset{¯}{\Delta \varphi (t)}=\overset{¯}{\varphi (t)}-\overset{¯}{\varphi (t=0)}$ of the average phase of each frame φ(t) with respect to the average phase of the initially acquired QPI image $\overset{¯}{\varphi (t=0)}$ was determined. The plot of the temporal development $\overset{¯}{\Delta \varphi (t)}$ is presented in Fig. 3(a). Figure 3(b) depicts the spatial distribution corresponding of the standard deviation (STD) $\sigma_{t}(m,n)=\frac{1}{300}\sqrt{\overset{300}{\underset{t=0}{\sum }}{\overset{¯}{\Delta \varphi (m,n,t)}}^{2}}$ that was calculated for each pixel of the entire stack of 300 QPI images. The corresponding histogram of the computed values $\sigma_{t}$ is depicted in Fig. 3(c). The mean value $\overset{¯}{\sigma_{t}}=0.07\;\mathrm{rad}$ indicates a high temporal phase stability.

### 3.3 Evaluation of QPI Accuracy Using Microspheres

**Fig. 4:**
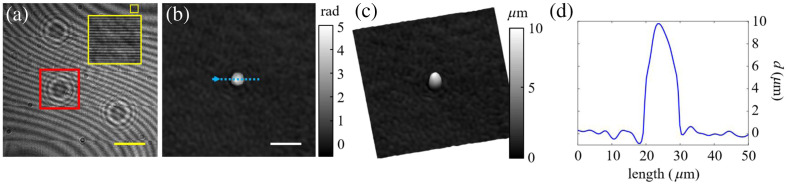
Validation of QPI image retrieval with LESSMIM by analysis of PMMA microspheres in a glycerol/water mixture (90%/10%). (a) Recorded off-axis hologram, (b) reconstructed numerically focused QPI image, (c) gray level coded pseudo-3D representation of thickness distribution computed from phase data in panel (b), and (d) thickness profile along the dotted blue line in panel (b). Yellow and white scale bars in panels (a) and (b) correspond to 100 and 20  μm, respectively.

The performance of LESSMIM for QPI was validated experimentally by analyzing PMMA microspheres with a diameter of 9.8  μm (PolyAn GmbH, lot number PT1140130FS) that were immersed in a mixture of glycerin and water (90%/10%). Figure 4(a) depicts a recorded digital off-axis hologram of 3  μA. The enlarged area of the yellow-framed region visualizes the off-axis carrier fringe pattern. Following the recovery process described in Sec. 3.1, the numerically focused QPI image of the microsphere enclosed in the red-framed square of Fig. 4(a) is obtained [Fig. 4(b)]. Here, a blank reference hologram was achieved by imaging a region of the sample without microspheres. Considering the refractive indices of the microspheres $n_{\mathrm{PMMA}}=1.494$ (Ref. 72) and the immersion liquid $n_{\text{medium}}=1.458$ (measured with an Abbe refractometer at λ=515  nm), the thickness distribution d(x,y)=Δθ(x,y)·λ/2πΔn of the microsphere was computed from the background-corrected phase distribution Δθ(x,y) of the QPI image in Fig. 4(b) and the RI difference $\Delta n=n_{\mathrm{PMMA}}-n_{\text{medium}}$ between the microspheres and the surrounding glycerol-water mixture.^73^ A pseudo-three-dimensional (3D) representation of the microsphere thickness distribution is presented in Fig. 4(c). Figure 4(d) shows a thickness profile along the dotted blue line in Fig. 4(b). To obtain the maximum thickness of the microsphere, we evaluated 10 data points around the center of the microsphere from which the mean value as well as the standard deviation were calculated. The obtained value $d_{\max}=9.7\pm 0.2\;\mu m$ for the maximum thickness matches the microsphere diameter of 9.8  μm provided by the manufacturer and validates LESSMIM for accurate QPI.

### 3.4 QPI of Living Adherently Grown Pancreatic Tumor Cells

**Fig. 5:**
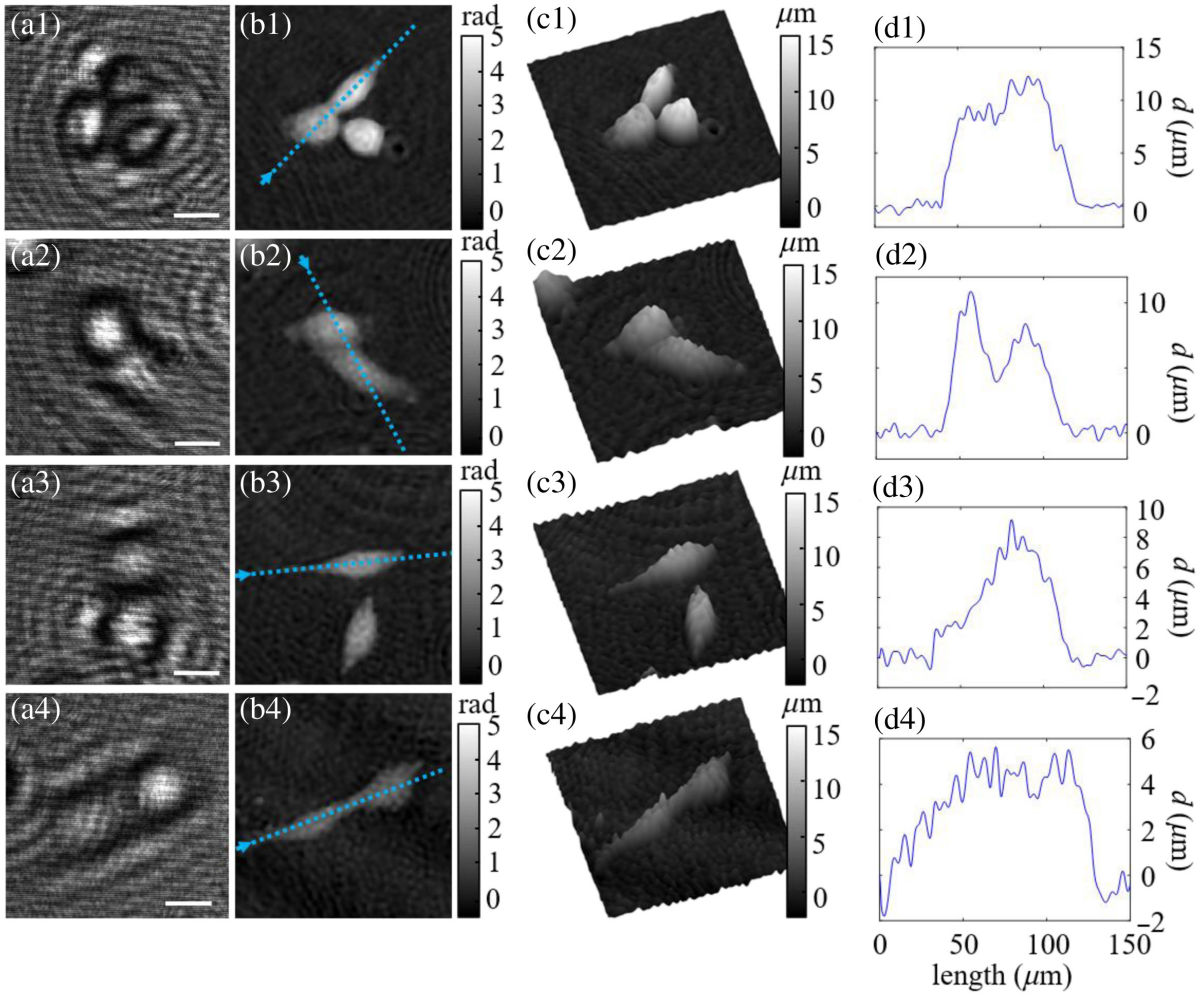
Evaluation of LESSMIM for QPI of living adherent pancreatic tumor cells (PaTu 8988T). Rows (1)–(4): ROIs containing cells with various morphologies and thicknesses. First column (a1–a4): recorded off-axis holograms; second column (b1–b4): reconstructed focused QPI images; third column (c1–c4): gray level coded pseudo-3D plots of the cell thickness distributions calculated from panels (b1–b4); and fourth column (d1–d4): thickness profile along blue dotted lines marked in panels (b1–b4). Scale bars in panels (a1–a4) correspond to 20  μm.

To assess the capability of LESSMIM for QPI of biological samples, investigations on living adherent pancreatic tumor cells (PaTu 8988T) were performed.^74^^,^^75^ Subconfluently grown cells were observed in Dulbecco modified Eagle’s medium (DMEM) between a glass object carrier slide and a cover slip (thickness of 175  μm). A blank reference hologram was achieved by imaging a sample region without cells. Figure 5 presents experimental results from adherent cells. Rows include different adherent cells exhibiting different morphologies and thicknesses. The first column (a1 to a4) in Fig. 5 displays the recorded off-axis holograms. In the second column (b1 to b4) of Fig. 5, the corresponding numerically focused QPI images are shown. Similar to the analyzed microspheres in Sec. 3.3, the cell thickness was determined by considering an average cellular refractive index of $n_{\mathrm{PaTuT}}=1.3654$^76^ and a refractive index of $n_{\mathrm{DMEM}}=1.339$ of the culture medium (measured by an Abbe refractometer). Pseudo-3D visualizations of the resulting cell thickness distributions are plotted in the third column (c1 to c4) of Fig. 5. Finally, Figs. 5(d1)–5(d4) include thickness profiles along the dotted blue lines in Figs. 5(b1)–5(b4). Additional results from isolated PaTu 8988T single cells are shown in Fig. S1 in the Supplemental Material. The experimental results demonstrate that LESSMIM successfully provided QPI images of both adjacent and isolated living adherent cells having different morphologies and axial dimensions, even for the case of thin adherent cells (as shown in row 4 of Fig. 5).

### 3.5 Quantitative Phase Imaging of Cells in Flow Within a Microfluidic Device

**Fig. 6:**
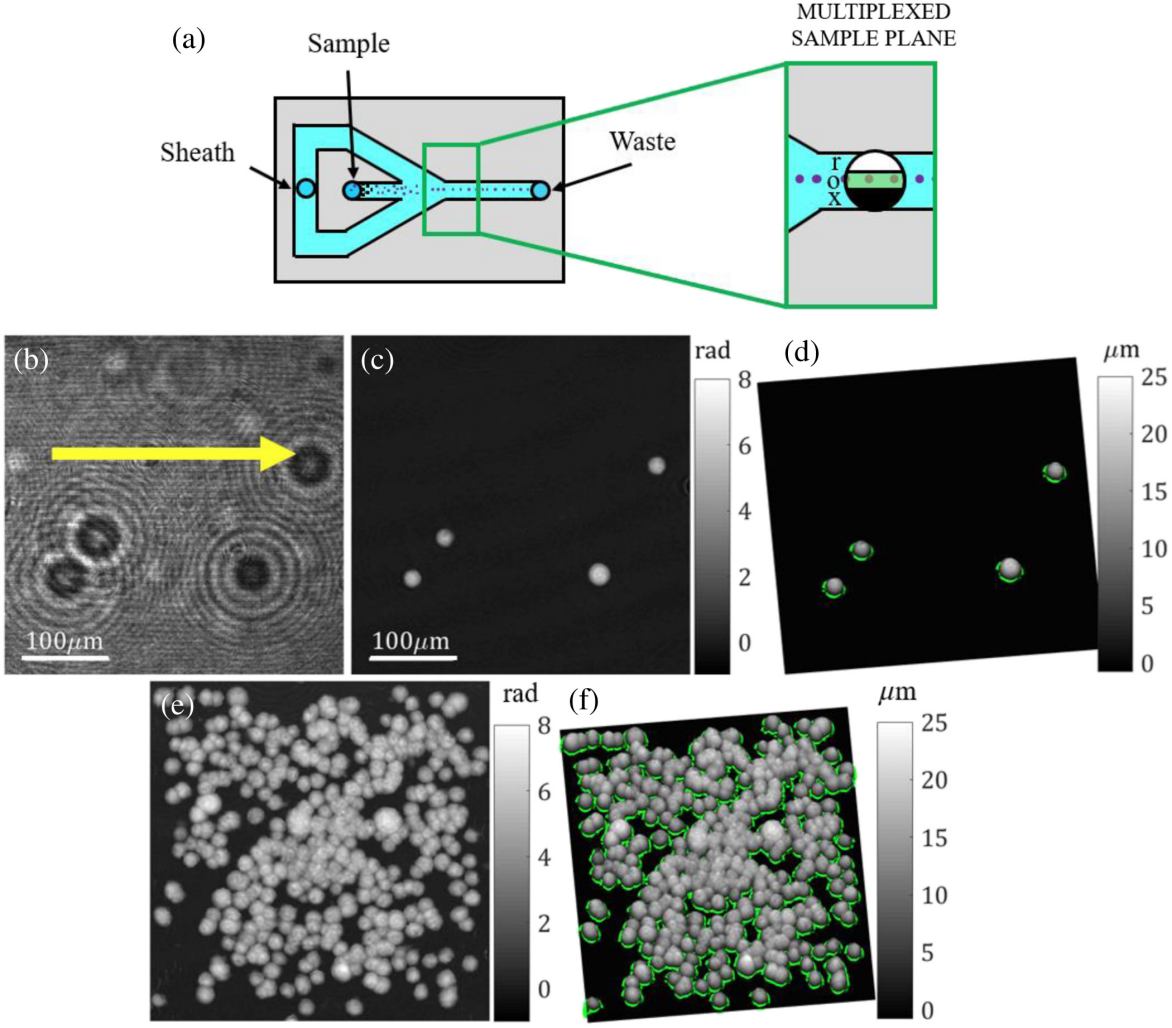
Evaluation of LESSMIM for IFC by investigations on living Patu 8988T cells in a microfluidic chip with hydrodynamic focusing capabilities. (a) Scheme of the utilized microfluidic chip with the enlarged region (green-framed rectangular) considered for lensless holographic imaging by spatial multiplexing (x is the blocking region, o is the object region, and r is the reference region). (b)–(d) Representative images of a recorded movie of 5 s that was recorded at a hologram acquisition rate of 40 fps with an exposure time of 2 ms (Video 1). (b) Representative off-axis hologram (the yellow arrow indicates the direction of the sample fluid stream); (c) focused QPI image reconstructed from panel (b); and (d) pseudo-3D thickness distribution computed from panel (c), with green outlines being the area retrieved by segmentation and indicating the cell boundaries. (e)–(f) Simultaneous visualization of all detected cells in the recorded QPI image stack achieved by maximum intensity projection (MIP); (e) MIP QPI image; and (f) corresponding pseudo-3D thickness representation with outlined cell boundaries. (Video 1, MOV, 7.67 MB [URL: https://doi.org/10.1117/1.JBO.29.S2.S22715.s1]).

The capability of LESSMIM for QPI of fast events and imaging flow cytometry (IFC) was evaluated by observation of living suspended PaTu 8988 T cells in a microfluidic chip within a rectangular cross-section (channel width of 1 mm and channel height of 20  μm) and hydrodynamic focusing capabilities as sketched in Fig. 6(a). The microfluidic device employed a syringe pump (Nemesys, CETONI GmbH, Korbußen, Germany) to direct the cells toward the microfluidic channel. The microfluidic channel was realized by standard soft lithography in polydimethylsiloxane (PDMS, Dow Corning, Midland, Michigan, United States), with a substrate that was 1 mm thick, and the PDMS was bonded on a glass coverslip (thickness of 175  μm) by an air-plasma activation as described in Refs. 77 and 78. To achieve lateral hydrodynamic focusing, the chip circuit was designed in co-flow architecture with an included inlet for splitting the sheath fluid into two separate fractions at the lateral channel borders and another inlet for the sample fluid in the center. The sheath fluid was introduced with two channels at an angle of 30 deg with respect to the channel for the sample fluid. The enlarged green-framed region in the right panel of Fig. 6(a) indicates the sample plane imaged by the LESSMIM setup and illustrates the need for hydrodynamic focusing to prevent the presence of cells within the reference area.

For the experiments, Patu 8988 T cells were suspended in DMEM at concentrations of $1.{5\cdot 10}^{6}\text{ cells}/\mathrm{ml}$ and observed with LESSMIM in a laminar flow at flow rates of 10  μl/min and 2  μl/min for the sheath fluid and the sample fluid, respectively. Off-axis holograms were recorded for 5 s at an acquisition rate of 40 fps and an exposure time of 2 ms. A blank reference hologram was recorded prior to the suspended cells passing through the microfluidic channel. The resulting holograms and QPI images are presented in Fig. 6 and Video 1. Figure 6(b) shows an exemplary off-axis hologram of a recorded image stack. The yellow arrow indicates the direction of the sample fluid stream. A representative-focused QPI image recovered from the hologram in Fig. 6(b) is depicted in Fig. 6(c). Figure 6(d) presents a pseudo-3D plot of the thickness distribution calculated from Fig. 6(c) by considering the same average RI value for PaTu 8988T cells as for the experiments in Sec. 3.4. Green outlines in Fig. 6(d) that were generated by the Otsu thresholding algorithm^68^ illustrate a reliable segmentation of cells in the QPI images and indicate the cellular boundaries. Figures 6(e) and 6(f) show images generated by maximum intensity projection (MIP) using Fiji,^79^ in which all cells that were detected in the recorded QPI image stack are visible. Figure 6(e) displays the combined image of the recovered focused QPI images, and Fig. 6(f) presents the corresponding image of the pseudo-3D thickness distributions with outlined cellular boundaries.

**Fig. 7:**
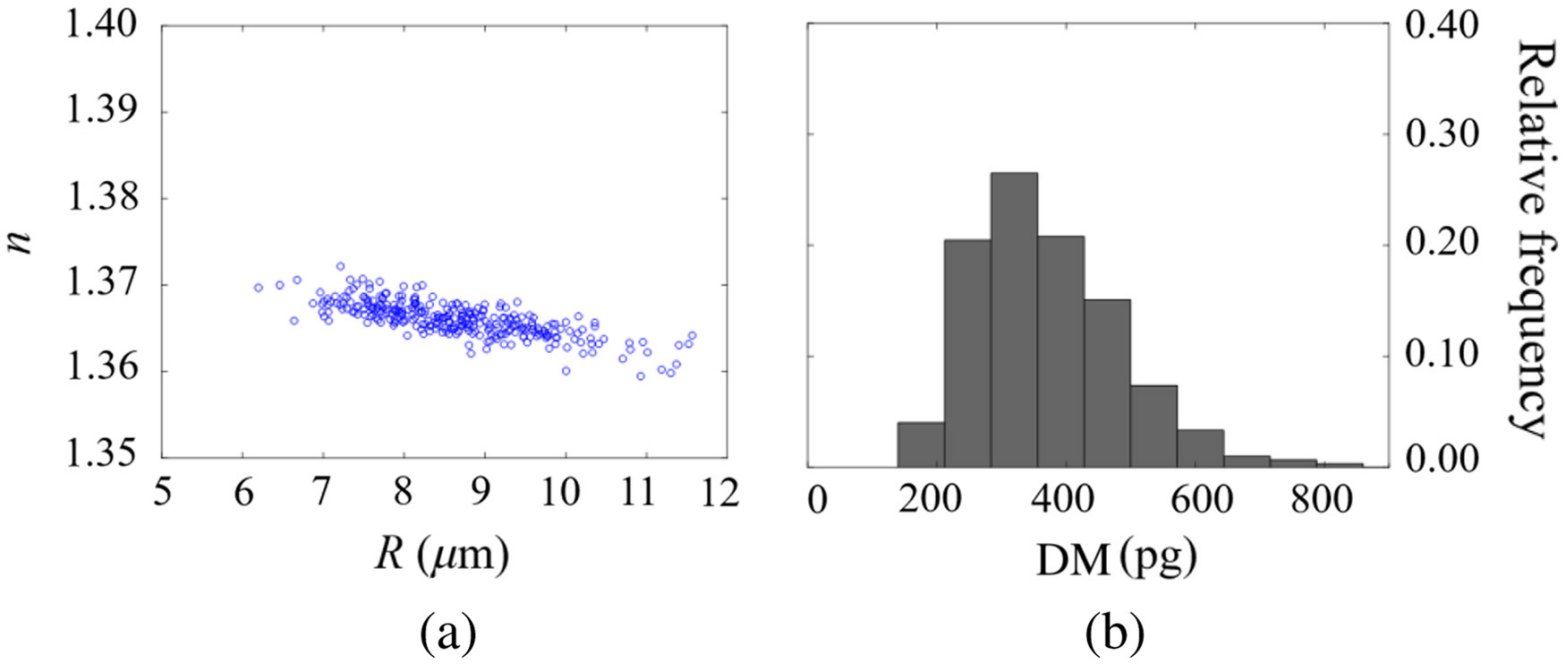
Determination of biophysical parameters from 300 individually analyzed suspended pancreatic tumor cells (Patu 8988T) in flow as illustrated in Fig. 6. (a) Scatter plot of the integral cellular RI n versus the projected cell radius R and (b) the relative frequency histogram of the dry mass DM.

To further evaluate the suitability of LESSMIM for IFC, biophysical features including the projected radius, integral refractive index, and cellular dry mass of the investigated cells in flow were determined from the recorded QPI image stacks. A total of 300 single cells (included in Video 1) were segmented and analyzed, whereas clustered cells in the respective images were discarded. Therefore, in the first step, segmentation was performed in each QPI image of a recorded stack, as illustrated in Fig. 6(c), to determine the projected area S of every analyzed single cell. Subsequently, assuming a spherical shape, as typical for suspended PaTu 8988 T cells,^75^ the cell radius R was calculated from the parameter S as $R=\sqrt{S/\pi }$. Moreover, as described in Refs. 75 and 76, the cellular dry mass $DM=10\lambda \overset{¯}{\Delta \varphi }S/2\pi \beta$ was calculated from the average phase value $\overset{¯}{\Delta \varphi }$ induced by the analyzed cell and the RI increment β of the intracellular content, which was estimated to be $0.002\;m^{3}/\mathrm{kg}$ for the investigated pancreatic tumor cell line.^80^ Finally, the integral RI $n=n_{\mathrm{DMEM}}+\overset{¯}{\Delta \varphi }\cdot \lambda /2\pi \overset{¯}{h}$ of the cells was computed, where $\overset{¯}{h}$ represents the mean cell thickness determined from QPI images of spherical cells as described in Refs. 75 and 76. Figure 7(a) shows the plot of the retrieved integral cellular RI n versus the corresponding projected cell radius R for the 300 analyzed cells, and Fig. 7(b) displays the relative frequency histogram of the corresponding dry mass DM values. The mean values $\overset{¯}{R}=8.64\pm 0.06\;\mu m$, $\overset{¯}{n}=1.366\pm 0.001$, and $\overset{¯}{\mathrm{DM}}=376\pm 7\;\mathrm{pg}$ are in good agreement with experimental data from previous investigations on PaTu 8988T cells.^76^

## 4 Discussion and Conclusions

In this study, we propose lensless spatially multiplexed interferometric microscopy (LESSMIM) as a single-shot DLHM technique to provide accurate QPI reconstruction and twin-image removal. LESSMIM is a simplified variant of the previously reported SMIM concept,^60^^,^^63^ in which imaging lenses are no longer required. LESSMIM comprises a common-path interferometric architecture that is based on a single diffraction grating to achieve digital off-axis holography. QPI of biomedical samples is achieved from single off-axis holograms using Fourier filtering and numerical propagation procedures. First, we characterized the technique concerning the lateral resolution (Fig. 2), temporal stability (Fig. 3), and QPI accuracy (Fig. 4) by utilizing a USAF resolution test target, a blank object carrier slide, and PMMA microspheres, respectively. Moreover, we demonstrated, for the example of pancreatic tumor cells, the capabilities of LESSMIM for QPI of living adherent cells (Fig. 5, Fig. S1 in the Supplemental Material) with different morphologies and thicknesses and for the retrieval of sets of biophysical parameters from fast-moving cellular specimens within a microfluidic channel with hydrodynamic focusing capabilities (Figs. 6 and 7).

LESSMIM offers several advantages over other DHM techniques. It utilizes a common-path interferometric architecture that leads to higher temporal stability and lower demands on the coherence properties of the utilized light source than double-path schemes.^64^^,^^81^^–^^83^ Although in our study, the geometry and the quality of optics in the illumination unit were selected to achieve a flexible experimental arrangement, LESSMIM permits a simpler, more compact, and more cost-effective design than typical Mach-Zehnder interferometer-based DHM arrangements.^8^^,^^14^^,^^15^^,^^84^ In particular, when LESSMIM is compared with common-path DHM systems such as diffraction phase microscopy,^82^ LESSMIM avoids the use and precise alignment of spatial filters, but at the cost of requiring a blank reference region near the specimen. Moreover, due to the spherical divergent wavefront employed for sample illumination, it enables variable magnification by simply changing the axial position of the point sources while maintaining spatial resolution.^70^ This characteristic allows for flexibility in the FOV size, which can be extended up to the size of the digital sensor for a magnification equal to 1 [see Eq. (3)], as in wide-field on-chip microscopy.^48^^,^^85^ With respect to previously reported in-line DLHM approaches, LESSMIM overcomes the limitation of the twin image presence and enables an accurate reconstruction of QPI images. Moreover, in comparison with earlier reported off-axis DLHM concepts that are based on two-pinholes, Fresnel biprism, a pair of GRIN lenses, or a prism with two-volume hologram gratings, the diffraction grating-based off-axis principle of LESSMIM offers a simplified arrangement, alignment, and handling; improved light transmission efficiency; and increased cost effectiveness.^56^^–^^59^ In addition, LESSMIM provides similar or even higher spatial resolution than previously reported off-axis DLHM approaches^56^^–^^59^ and an FOV comparable to or slightly higher than that described in previous works.^56^^,^^59^ Regarding accuracy and temporal stability, only a few previously reported related approaches^58^^,^^59^ analyzed these features. In this study, we demonstrated that LESSMIM provides accurate QPI images and a temporal stability at an observation period of 5 min that is comparable to previously reported approaches.^58^^,^^59^

However, LESSMIM also has limitations. In LESSMIM, an object-free region near the sample is required, which limits the analysis of extended samples, as happens in lens-based SMIM systems.^60^^,^^63^ However, this issue can be addressed by customized micropatterned sample carriers.^86^ When LESSMIM is combined with a microfluidic system for IFC, this drawback can be overcome by either designing a microfluidic channel with a small width or, as in our study, employing microfluidic systems with hydrodynamic focusing. The spatially multiplexed approach also reduces the object region to one third of the illuminated FOV. However, in practice, this limitation is not significant as the illuminated sample region typically is larger than the recorded FOV. Another limitation of LESSMIM is the recording of out-of-focus holograms because it requires numerical propagation algorithms to achieve sharply focused QPI images, which increases the computational amounts and prevents correlative imaging with other modalities, such as fluorescence or bright field microscopy. Moreover, the system introduces coma aberration, which can be compensated for by either recording an additional reference hologram (as shown in Fig. 2), using various established state-of-the-art computational methods^87^^,^^88^ such as the utilization of Zernike polynomials,^89^ or more recent approaches such as principal component analysis^90^ or deep learning,^91^ etc.^92^^,^^93^ It is worth noting that this issue is not critical for the application of LESSMIM to IFC because a reference hologram can be recorded at the beginning of the experiment when no sample fluid is present inside the microfluidic channel. When compared with state-of-the-art DLHM systems, the LESSMIM setup presents relatively large axial dimensions (around 200 mm), but it can be further reduced by optimization of the axial distances (lenses with lower focal lengths and/or a smaller separation between lenses).

In summary, LESSMIM has been demonstrated to be a compact and cost-effective method for accurate QPI that is also capable of imaging fast biomedical events. Future developments include the further miniaturization of the setup, investigations on optical components for further cost reduction, and methods for digital correction of phase aberrations. In particular, we will investigate changing the microscope objective, utilized in our study for illumination focusing, to a more cost-effective lens with a high numerical aperture for which additional aberrations can be assumed and that can be addressed numerically by adapted aberration correction algorithms. In addition, investigations to further reduce optical elements and space within the illumination unit will be performed to minimize the dimensions of the LESSMIM setup and to increase the cost effectiveness. LESSMIM will be useful for applications in various biomedical fields, particularly those for which analysis of fast-moving samples is required such as imaging flow cytometry^94^ or sperm assessment,^25^ as it is a DHM technique particularly valuable for point-of-care diagnosis and laboratories with limited budgets.

## Supplementary Material





## Data Availability

Data underlying the results presented in this article are available by a reasonable request to the corresponding authors.
