# Supplementary material for: Off-axis digital lensless holographic microscopy based on spatially multiplexed interferometry
Source: J Biomed Opt. 2024 Aug 19;29(Suppl 2):S22715. doi: 10.1117/1.JBO.29.S2.S22715 (PMC11331263; doi:10.1117/1.JBO.29.S2.S22715)
Supplement: Supplementary file 1 [file JBO_029_S22715_SD001.pdf]

Off-axis digital lensless holographic microscopy based on spatially-multiplexed interferometry

José Ángel Picazo Bueno,[a,b,*] Steffi Ketelhut,[a] Jürgen Schnekenburger,[a] Vicente Micó[b], Björn Kemper[a,*]
[a]University of Muenster, Biomedical Technology Center, Mendelstr. 17, D-48149 Muenster, Germany
[b]University of Valencia, Department of Optics, Optometry and Vision Science, c/ Dr. Moliner 50, 46100 Burjassot, Spain

Keywords: quantitative phase imaging, off-axis lensless holography, digital lensless holographic microscopy, label-free imaging, digital holographic microscopy, phase retrieval, spatially multiplexed interferometric microscopy.

*****Corresponding authors: José Ángel Picazo-Bueno, E-mail: picazobu@uni-muenster.de; Björn Kemper, E-mail: bkemper@uni-muenster.de

S1 Alignment protocol of the instrument

"The alignment of the system sketched in Fig. 1 of the main text is performed similar to a common bright field microscope. The fiber-coupled laser diode is located in the optical axis and in the object focal plane (f_{CL}) of the collimating lens. Then, the focusing lens is placed at a certain distance from the collimating lens to generate a spherical wave front. Subsequently, the sample and the digital sensor are positioned at the distances $f'_{FL} + z_1$ and $f'_{FL} + z_1 + z_2$, from the focusing lens to achieve the desired imaging performance in terms of resolution, magnification and FOV. Note that the magnification of the system can be modified by axial displacement of the focusing lens along the optical axis, which changes also the plane in which the point sources are generated. Finally, the diffraction grating is inserted into the path of the collimated illumination between collimating lens and focusing lens to generate three replicas of the point source and to achieve an off-axis holography configuration. Note that in the alignment process, an opaque edge of the grating frame was employed to block one third of the beam to avoid spurious interferences."

S2 QPI of single isolated living adherently grown pancreatic tumor cells

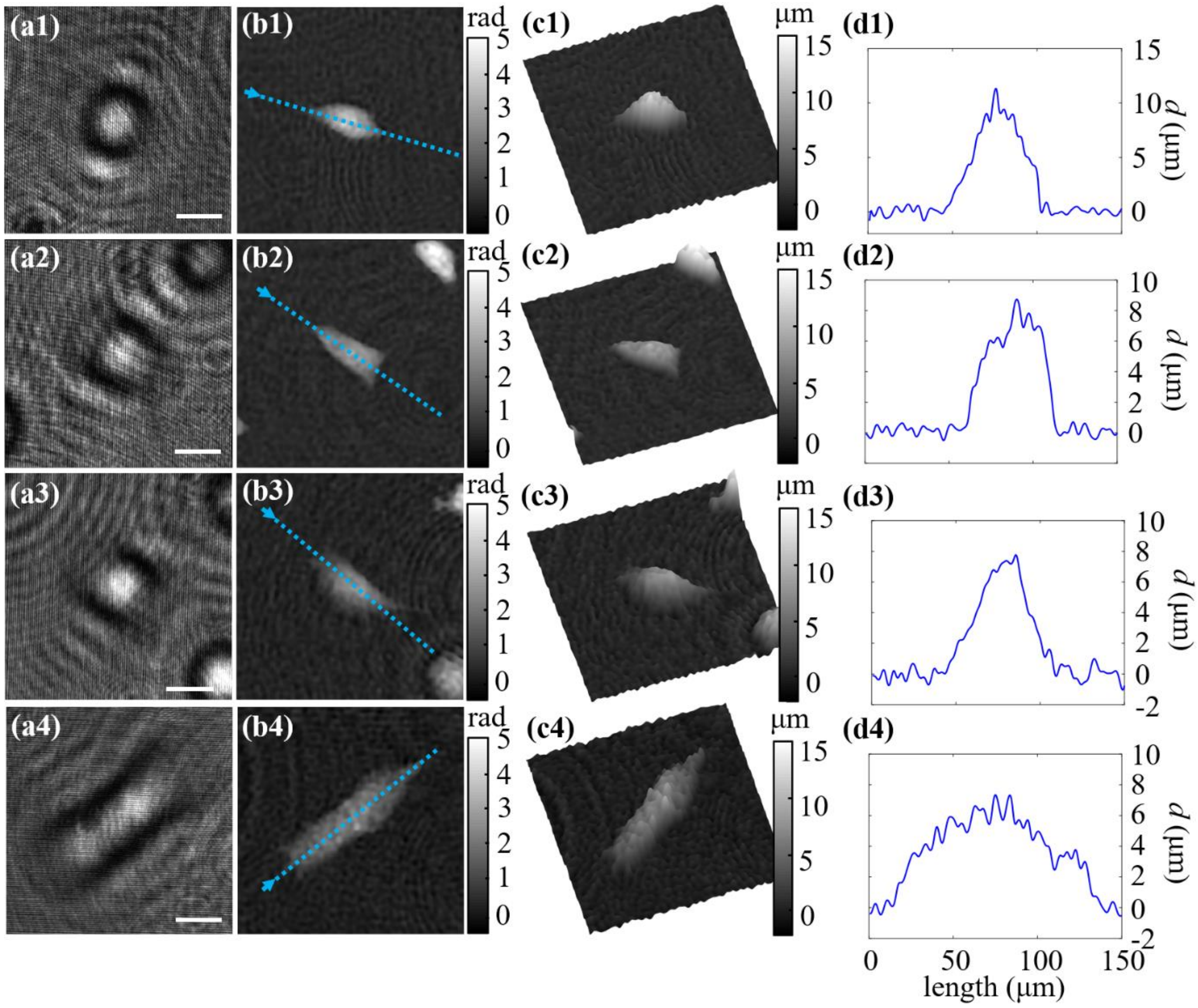

Fig. S1 Evaluation of LESSMIM for QPI of single isolated living adherent pancreatic tumor cells (PaTu 8988T). Rows (1)-(4): ROIs containing single isolated cells with various morophologies and thicknesses. First column (a1-a4): recorded off-axis holograms; second column (b1-b4): reconstructed focused QPI images; third column (c1-c4): gray level coded pseudo 3D plots of the cell thickness distributions calculated from (b1-b4); fourth column (d1-d4): thickness profile along blue dotted lines marked in (b1-b4). Scale bars in (a1-a4) correspond to 20 µm.
